# Factors associated with long-acting family planning service utilization in Ethiopia: a systematic review and meta-analysis

**DOI:** 10.1186/s40834-019-0095-z

**Published:** 2019-10-01

**Authors:** Tamirat Tesfaye Dasa, Teshager Worku Kassie, Aklilu Abrham Roba, Elias Bekele Wakwoya, Henna Umer Kelel

**Affiliations:** 1 Reproductive Health and Maternal, School of Nursing and Midwifery, College of Health and Medical Sciences, Haramaya University, P.O. Box 235, Harar, Ethiopia; 2 College of Medicine and Health Sciences, Arba Minch University, Arba Minch, Ethiopia

**Keywords:** Long-acting contraceptive, Married women, Ethiopia, Systematic review and meta-analysis

## Abstract

**Background:**

Even though the modern contraceptive use was improved in Ethiopia, the utilization of long-acting family planning services is still low because of numerous factors. The aim of this systematic review was to synthesize logical evidence about factors associated with long acting family planning service utilization in Ethiopia.

**Methods:**

The participants of the study were married women of reproductive age in Ethiopia. This search included all published and unpublished observational studies written in the English language conducted before April 30, 2018, in Ethiopia. Electronic and non-electronic sources were used. PubMed, MEDLINE (EBSCO), CINHAL (EBSCO), Embase (EBSCO), POPLINE and the search engines like Google, Google Scholar Mednar and world cat log were used. The overall selected search results were 15 studies. Each study was evaluated using the Joanna Briggs Institute Quality Assessment Tool for Observational Studies. Data synthesis and statistical analysis were conducted using ReviewManagerVersion5.3.5.

**Results:**

Women’s inadequate knowledge level [OR, 0.29; 95% CI: 0.10, 0.83, *P* = 0.02], women’s age between 15 and 34 [OR, 0.82; 95% CI: 0.53, 0.93, *P* = 0.01], not having electronic media [OR, 0.65; 95% CI: 0.53, 0.79, *P* < 0.0001] and women from rural area [OR = 0.65;95% CI:0.50, 0.81, *P* = 0.0009] were less likely associated in the use of long-acting family planning services. The odds of utilizing long acting family planning methods were high among non-government- employed women and husband [OR, 1.77; 95% CI: 1.29, 2.43, *P* = 0.0004], [OR, 1.69; 95% CI: 1.33, 2.15, *P* < 0.0001] respectively. Having no previous exposure to any modern family planning method [OR = 2.29; 95%CI: 1.83, 2.86, *P* < 0.00001] and women having no discussion with husband [OR = 1.92 (95%CI: 1.50, 2.45) P < 0.00001] were more likely associated in the utilization of long-acting family planning services.

**Conclusion:**

Lack of information and knowledge, having discussion with husband, being women of younger age, having less than five living children, being government-employed women and husband, not having electronic media, and being residents in rural area were significant barriers for underutilization of long acting family planning methods in Ethiopia. Hence, the investigators suggest that key stakeholders should design interventions strategies to avert attitudinal, cultural and informational barriers towards long-acting family planning methods.

**Systematic review registration:**

PROSPERO: 2018: CRD42018096373.

The online version of this article (10.1186/s40834-019-0095-z) contains supplementary material, which is available to authorized users.

## Introduction

The modern contraceptive use has steadily increased over the past 15 years in Ethiopia. The contraceptive prevalence rate in 2000 was only 6.3%, which accelerated to 35% in 2016. However, utilization of long-acting family planning methods (LAFPM) is still low compared to the injectable contraceptives [1, 2]. According to the Ethiopian Demographic and Health Survey (EDHS) 2016 Report the most commonly used contraceptive method for currently married women in Ethiopia are injectable (23%) followed with implants (8%). The total fertility rate in Ethiopia is 4.6 children per woman and 22% percent of currently married women have an unmet need for family planning. Shifting towards LAFPM is the best strategy to ensure continuity of the family planning service in a country like Ethiopia where there is high fertility rate and unmet need for family planning [2].

Long-acting family planning methods (LAFPM) can be permanent or reversible; are methods that prevent pregnancy more than three years per application which include subdermal implants, intrauterine devices (IUD) and male and female sterilizations. These methods have many advantages compared to other family planning methods. They are convenient, very effective, long-lasting, reversible and cost-effective. In addition to these the effectiveness of LAFPM are not dependent on compliance with taking the oral contraceptives daily or taking the regular injection at clinics; therefore they prevent the failure rate due to the incorrect use [3]. It is estimated that 1,250 unwanted pregnancies would have prevented if 5000 oral contraceptive users were to switch to intrauterine device or implants over a period of years [4].

Different pocket primary studies were conducted in different parts of Ethiopia to determine the factors associated with utilization of long-acting family planning methods (LAFPM). There are few comprehensive studies done in Ethiopia about LAFPM. The study conducted by Yonatan et al. assessed the practice and intention to use long-acting and permanent contraceptive methods among married women in Ethiopia by using systematic review and meta-analysis [5]. However, this study did not address the factors associated with the utilization of long-acting contraceptives in Ethiopia. Therefore, the aim of this study is to summarize the evidence of factors associated with LAFPM utilization among married women in Ethiopia. The summarized evidence obtained from this study helps the concerned bodies to identify existing gaps and propose strategies to increase the utilization of long-acting family planning methods (LAFPM) in Ethiopia.

## Methods

### Protocol and registration

This review was developed based on the PRISMA (Preferred Reporting Items for Systematic Reviews and Meta-Analyses) guideline [6](See Additional file 1). The review has been registered protocol by the International prospective register of systematic reviews (CRD42018096373).

### Eligibility criteria

The study participants were married women of reproductive age in Ethiopia. They were from all socio-economic status, all ethnic groups and language. This search included all published and unpublished observational (cross-sectional and case-control) studies on factors affecting long-acting family planning service utilization among married women of reproductive age in Ethiopia. It included studies conducted before April 30, 2018 and were written in the English language. Reviews, commentaries, editorial, case series/report, and patient stories were excluded from the systematic review process.

### Information source sand search strategy

This systematic review and meta-analyses is conducted according to PRISMA (Preferred Reporting Items for Systematic Reviews and Meta-Analyses) guideline [6]. The investigators retrieved information from electronic and non-electronic database sources. Electronic database sources: PubMed, MEDLINE (EBSCO), CINHAL (EBSCO), Embase (EBSCO) and POPLINE were used to retrieve published articles. Non-electronic sources used; direct Google search, Google Scholar, Mednar and world cat log.

Combination of search terms were used with (AND, OR, NOT) Boolean (Search) Operators. The search strategy included the use of Title/Abstract related to: (“family planning service” “AND” “Factors associated” or “Determinants” or “Predicators” “AND” Ethiopia) taken from the review questions. Non-electronic sources used were combined with direct Google search, Google Scholar, Mednar and worldcat log. In addition, the investigators searched manually for grey literature and other relevant data sources such as email and unpublished thesis/papers with planned dates of coverage. The search strategy for CINHAL is outlined in (Additional file 2).

### Study selection

All search articles were exported to the EndNote X8 citation manager and duplicated studies were excluded. Then studies were screened through by careful reading of the title and abstract. The three authors (TT, EB and TW) screened and evaluated studies independently. The titles and abstracts of studies that clearly mentioned the outcomes of the review (factors associated//determinants/predictors/ of family planning service utilization) were considered for further evaluation to be included in the systematic review and meta-analysis. Then the full-text of the studies were further evaluated based on objectives, methods, participants/population and key findings (Factors associated/affecting/determinants/predicators/long-acting family planning methods utilization. The two authors (AA and HU) independently evaluated the quality of the studies against the checklist. Any discrepancy was resolved through discussion or through asking a third reviewer if consensus could not be reached.

**Fig. 1:**
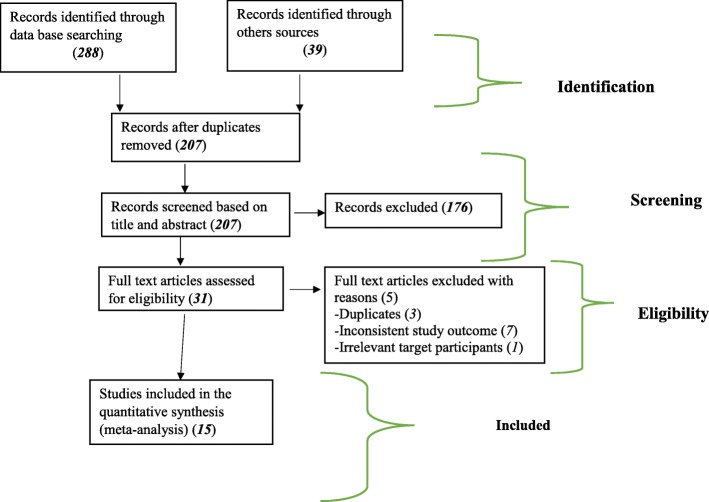
Description of schematic presentation of the PRISMA flow diagram to select and include studies, 2018

The overall study selection process is presented using the PRISMA statement flow diagram [7] (Fig. 1).

### Data collection process

After the selection of appropriate articles, data were extracted by two investigators independently (TW and EB) using a data extraction template and presented through Microsoft word 2016 (containing author & year, setting, study design, sample size, study subject, data collection methods, primary outcome of interest and specific factors associated with LAFPM utilization) (Table 1)*.* The accuracy of the data extraction was verified by comparing the results with the data extraction by the second three investigators (TT, AA, HU), who independently extracted the data in a randomly- selected subset of papers (30% of the total).

**Table 1:** Description of study participants and characteristics of Studies included in the systematic review and meta-analysis

No.	Authors & years	Setting of the study	Design of the study	Sample size	Study subject	Data collection methods	Primary outcome of interest	Specific factors associated with utilization of long-acting family planning methods (LAFPM) utilization
1.	Alemayehu, M. et al. 2012 [8]	Community-based	Cross-sectional	460	Married women of reproductive age	Structured Interviewer	Utilization of long-acting and permanent contraceptive methods (LAPCM)	- Knowledge, Number of pregnancies, Desire for more children
2.	Bulto, G. A. et al. 2014 [9]	Community-based	Cross-sectional	519	Married women of reproductive age	Structured interview	Demand forLAPCM	Age, Desire for more child, Duration of desire for a child, Number of children ever born, Discussion with a partner on a method to use
3.	Gebre-Egziabher, Dest et al. 2017 [10]	Community-based	Cross-sectional	524	Women of reproductive age	Structured interview	Utilization of Implant	Employment, Number of methods ever used
4.	Gebremariam, A. &Addissie, A. 2015 [11]	Community-based	Cross-sectional	591	Married women	Structured interview	Intention to use LAPCM	Partner’s education, Participant occupation, wants more child within 2 years, Know LAPCMs, Husband support LAPM use
5.	Gebremichael et al. 2015 [12]	Community-based	Cross-sectional	342	Married women	Structured interview	Acceptance of LACM	The attitude of respondent towards Acting Reversible Contraceptive acceptance
6.	Guidance, ShimelsWudie et al. 2015 [13]		Unmatched Case-control	360	Married Women	Interview	Use of Long-Acting Reversible Contraceptive	Age of respondent, Occupation, Husband-wife discussion
7.	Medhanyie, A. et al. 2017 [14]	Community-based	Cross-sectional	540	women of reproductive age group	Structured interview	Use of long-acting family planning	Residence
8.	Mekonnen, Getachew et al. 2014 [15]	Community-based	Cross-sectional	763	Women of reproductive age	Structured interview	Use of long-acting and permanent contraceptive	Age, Knowledge of Long-acting and permanent contraceptive methods
9.	Melka, A. S. et al. 2015 [16]	Community-based	Cross-sectional	1003	Married women of reproductive age groups	Structured interview	Utilization of long-acting and permanent contraceptive methods	Education, Occupation, Number of live children, the Joint decision on fertility with a partner, Have radio/TV
10.	Meskele and Mekonnen 2014 [17]	Community-based	Cross-sectional	416	Women	Structured interview	Use long-acting and permanent contraceptive	Educational status, Attitude score (composite), Heard myths and misconceptions
11.	Serawit, L. and Alemayehu, W. 2012 [18]	Facility-based	Unmatched Case-control	270	women of reproductive age	Structured interview	Use of the intrauterine contraceptive device	Educational status, Age of youngest child, IUCD causes infection
12.	Shiferaw, K. and Musa, A. 2017 [19]	Facility-based	Cross-sectional	400	women of reproductive age group	Structured interview	Utilization of long-acting reversible contraceptive	Occupation of woman, Ethnicity, Religion
13.	Tamrie, YirgaEwnetu etal 2015 [20]	Community-based	Cross-sectional	441	Mothers in the Postpartum Period	Structured interview	Use of Long-acting Reversible Contraception	Education of participant, Previous use of long-acting reversible contraceptive, Counseled on long-acting reversible contraceptive
14.	Yalew, SaleamlakAdbaruet al 2015 [21]	Facility-based	Cross-sectional	487	Women users of family planning	Structured interview	The demand for long-acting contraceptive users	Occupational status, Number of children, Number of discussions with husband, decision maker to use
15.	Zenebe, C. B. et al. 2017 [22]	Facility-based	Cross-sectional	317	women reproductive age group	Structured interview	Utilization of long-acting and permanent contraceptive	Educational status, Information about long-acting permanent contraceptive methods (LAPCM), Previous use of LAPCM

The quantitative data (the total sample size (n) and specific factors associated with utilization of long-acting family planning methods) were extracted from the included articles and summarized using Microsoft Excel 2016 for meta-analysis and synthesis.

### Data items

The determinants of long-acting family planning methods (LAFPM) utilization were the main outcome variables which were achieved by this systematic review and meta-analysis. The outcome variables were measured either by a direct report from the included studies or indirectly based on the statistics reported in the individual studies. To quantify the outcome “Factors associated with long-acting family planning methods utilization” the investigators considered studies which reported as determinants of LAFPM in their statistics differently and specifically, women’s knowledge level, women’s age; women’s education level; husband’s education level; number of living children; joint husband-wife discussion; women’s occupation; husband’s occupation; the presence of media; the residence of setting; previous history of utilizing family planning method and others to comprehensively quantify the determinants. The result was interpreted by odds ratio.

### Risk of bias in individual studies

Investigators critically evaluated the risk of bias from individual studies using the Joanna Briggs Institute Quality Assessment Tool for observational studies. To minimize the risk of bias comprehensive searches (electronic/database search and manual search) were used and also included published, unpublished facility or community-based studies and thesis. Cooperative work of the authors was also critical in reducing bias, in setting a schedule for the selection of articles based on the clear objectives and eligibility criteria, deciding the quality of the article, in regularly evaluating the review process, and in extracting and compiling the data. Publication bias was explored using visual inspection of the funnel plot. Besides, Egger’s Regression Test was carried out to check statistically symmetry of the funnel plot [23].

### Synthesis of data

Data synthesis and statistical analysis were conducted using Review Manager (RevMan) version 5.3.5. A meta-analysis of observational studies was carried out, based on the recommendations of the I^2^ statistic described by Higgins et al. (an I^2^ of 75/100% and above suggesting considerable heterogeneity). The investigators checked for potential publication bias through visual inspection of a funnel plot, and Egger’s Regression Test. Publication bias was assumed for *P*-values of less than 0.10. The results of the review were reported according to the PRISMA guidelines. The findings of the included studies were first presented using a narrative synthesis and followed by meta-analysis chart.

## Result

### Description of review studies

A total of 327 articles were identified through the major medical and health electronic databases and other relevant sources. From all identified studies, 120 articles were removed due to duplication while 207 studies were reserved for further screening. Of these, 176 were excluded after being screened according to titles and abstracts. Of the 31 remaining articles, 16 studies were excluded due to inconsistency with inclusion criteria set for the review. Finally, 15 studies which fulfilled the eligibility criteria were included for the systematic review and meta-analysis. General characteristics and descriptions of the studies selected for the meta-analysis were outlined in (Table 1)*.*

### Factors associated with utilization of long-acting family planning services

The results of this review have shown many factors associated with long-acting family planning services utilization in Ethiopia. Significant associated factors were the woman’s knowledge level, woman’s age, woman’s education level, husband’s education level, number of living children, joint husband-wife discussion, woman’s occupation, husband occupation, the presence of media, the residence of setting, and previous history of using family planning method. The review also verified that income was not a significant predictor of long-acting family planning services utilization.

### Woman’s knowledge level on family planning

**Fig. 2:**
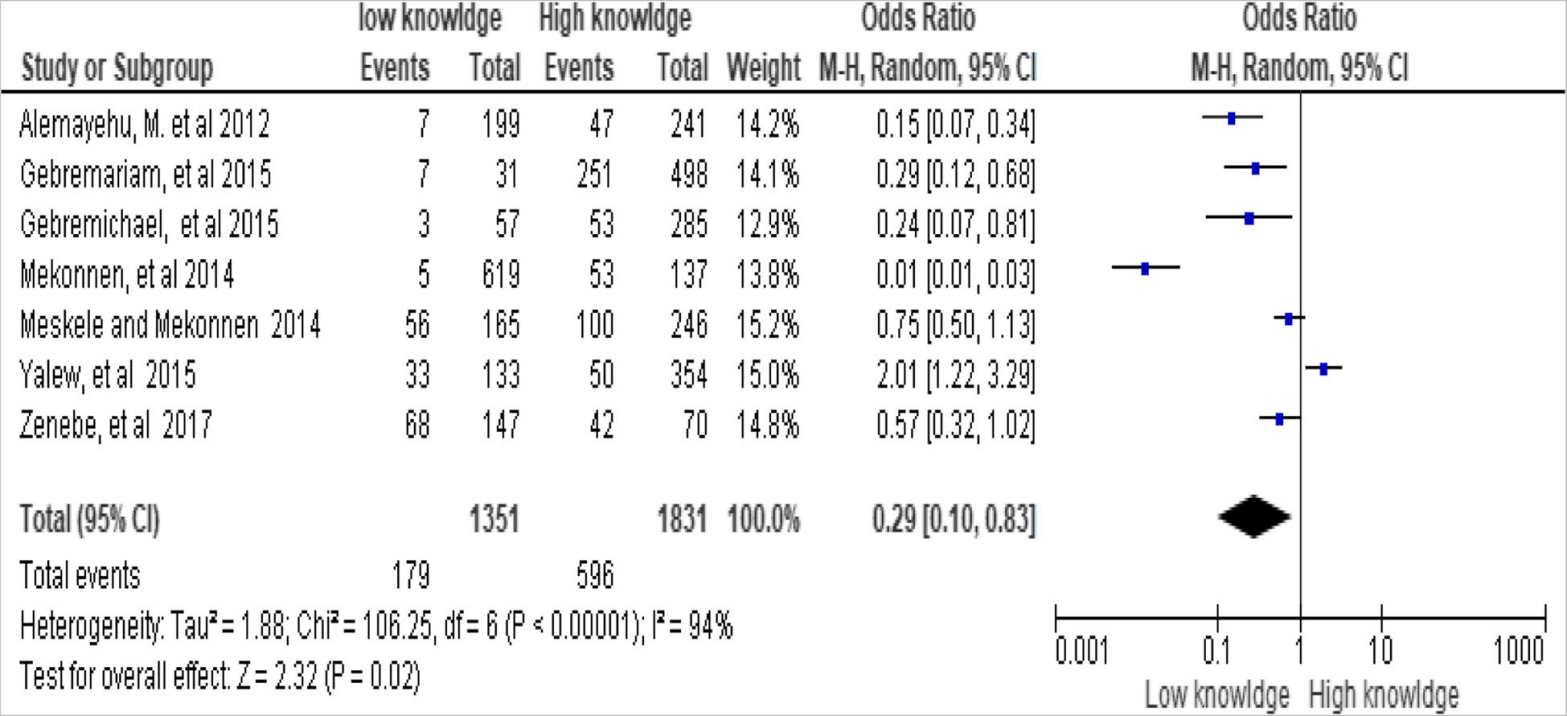
Association between woman’s knowledge levels with utilization of long-acting family planning services in Ethiopia, 2018

The level of woman’s knowledge was significantly associated with long-acting family planning services utilization. Women who had inadequate knowledge on modern family planning were less likely to utilize long acting family planning services compared to women who had adequate knowledge [OR, 0.29; 95% CI: 0.10, 0.83, *P* = 0.02]. Heterogeneity test indicated I^2^ = 94%, hence the random and fixed effect model was employed interchangeably for analysis. In addition, a sensitivity analysis was done, and no change was illustrious in the overall odds ratio (OR) (Fig. 2).

### Woman’s age

**Fig. 3:**
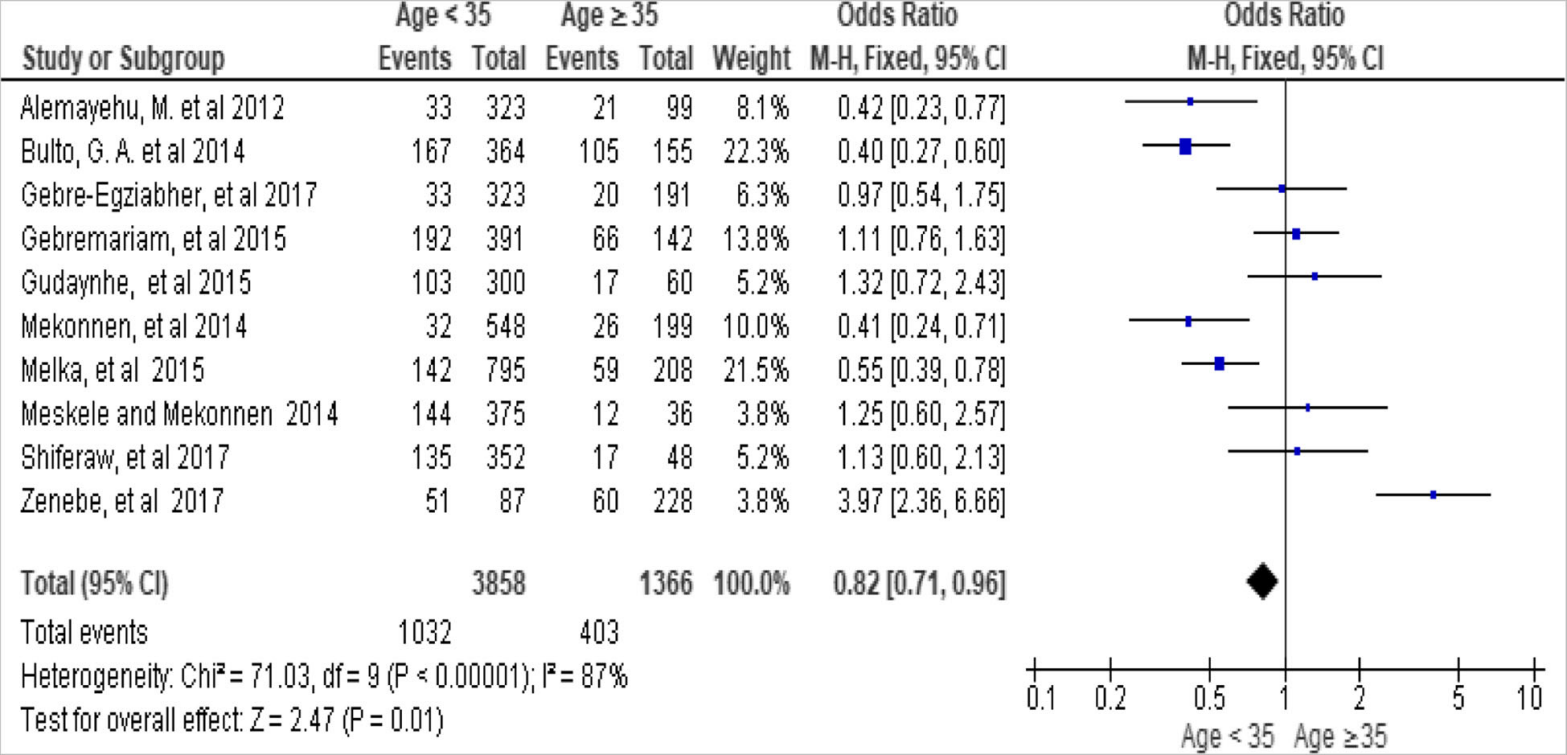
Association between woman’s age with long-acting family planning services utilization in Ethiopia, 2018

The woman’s age was significantly associated with utilization of long-acting family planning services. The odds of utilizing long acting family planning services were low among women 15 to 34 years of age as compared to those aged between 35 to 49 years [OR, 0.82; 95% CI: 0.53, 0.93, *P* = 0.01]. Heterogeneity test indicated *I*^2^ = 87%, hence random and fixed effect model was employed interchangeable for analysis, but no significant change on heterogeneity in both models. So, the investigators assume to employ fixed effect model for analysis because there was a small change in overall summary results (Fig. 3)*.*

### Woman’s occupation status

**Fig. 4:**
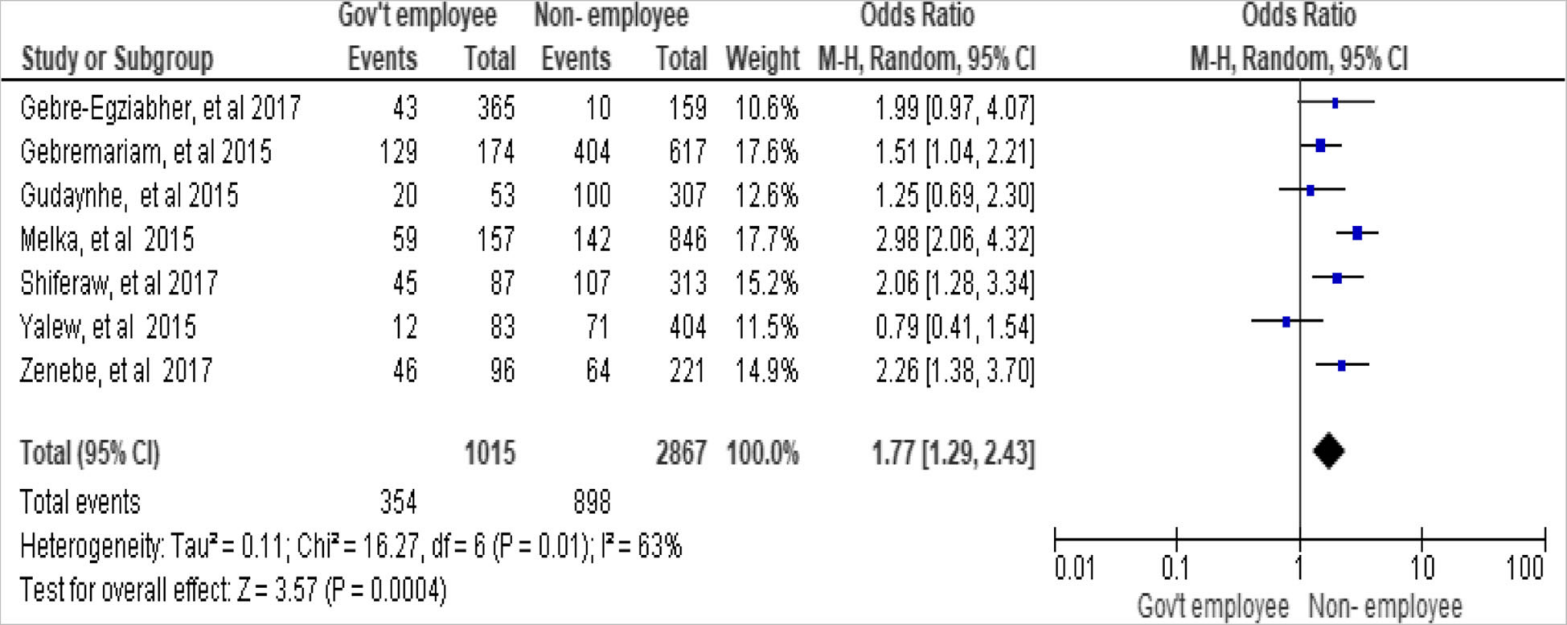
Association between woman’s occupations with utilization of long-acting family planning services in Ethiopia, 2018

An Odds Ratio revealed that there was a significant association between woman’s occupation and utilization of long-acting family planning services [OR, 1.77; 95% CI: 1.29, 2.43, *P* = 0.0004]. The non-government employed woman was 1.8 times more likely to have long-acting family planning services as compared to government- employed woman (Fig. 4)*.*

### Husband’s occupation status

**Fig. 5:**
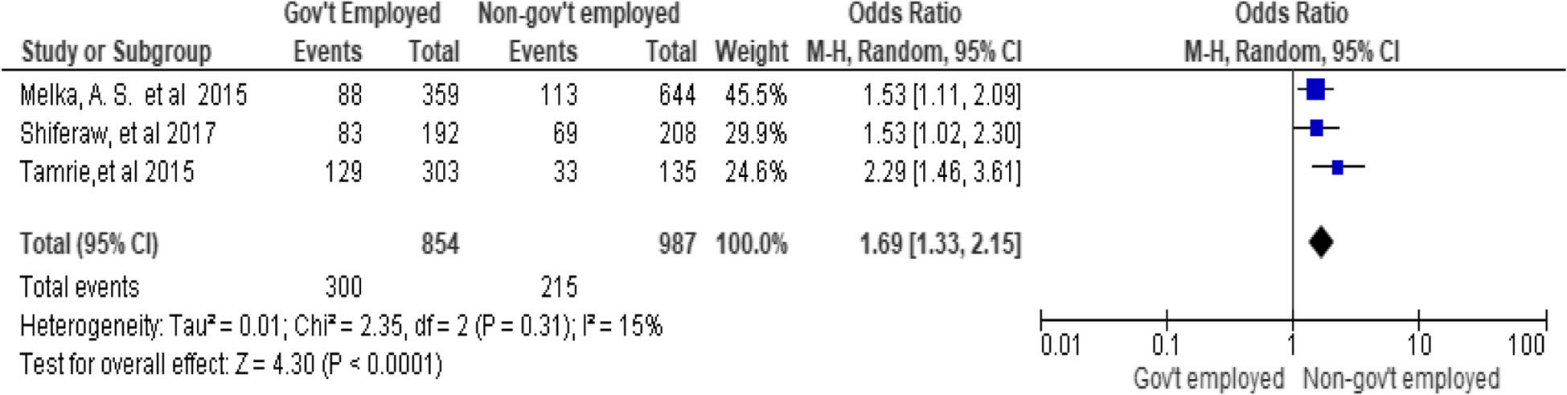
Association between husband occupations with long-acting family planning service utilization in Ethiopia, 2018

The results of the review showed statistically significant association between husband’s occupation and women’s utilization of long-acting family planning services. Women whose husbands were non-government employees utilize LAFP services than those whose husbands were government employees (OR, 1.69; 95% CI: 1.33, 2.15, *P* < 0.0001). The heterogeneity test indicated an I^2^ value of 15% (Fig. 5)*.*

### Women’s education level

**Fig. 6:**
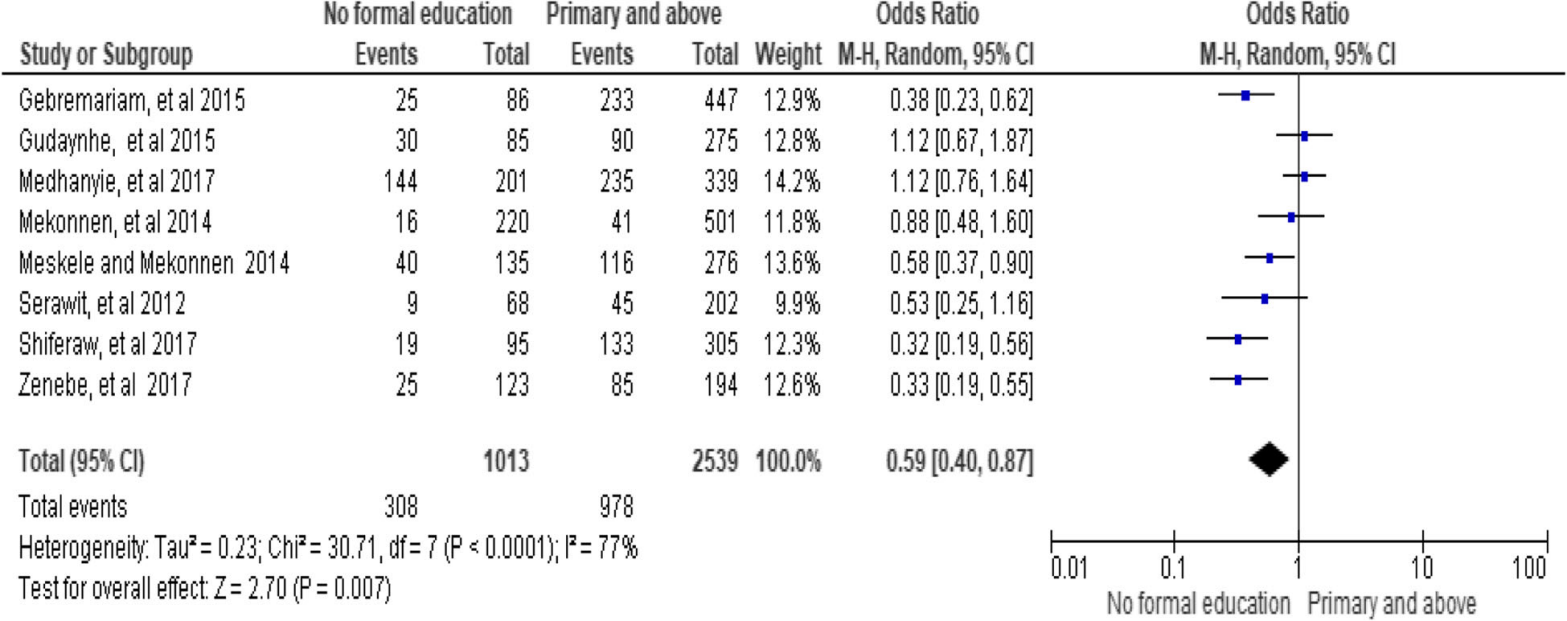
Association between women’s education with long-acting family planning service utilization in Ethiopia, 2018

The findings of the review indicated a significant association between women’s education level and utilization of long-acting family planning services. Women who have no formal education were 0.59 times less likely to utilize long-acting family planning services as compared to women who had primary education and above [OR = 0.59;95% CI: 0.40, 0.87, *P* = 0.007]. Heterogeneity test indicated *I*^2^ = 77%, hence random effect model was assumed in the analysis. Sensitivity was done of analysis but did not bring significant change in the overall summary results of OR (Fig. 6)*.*

### Husband’s education level

**Fig. 7:**
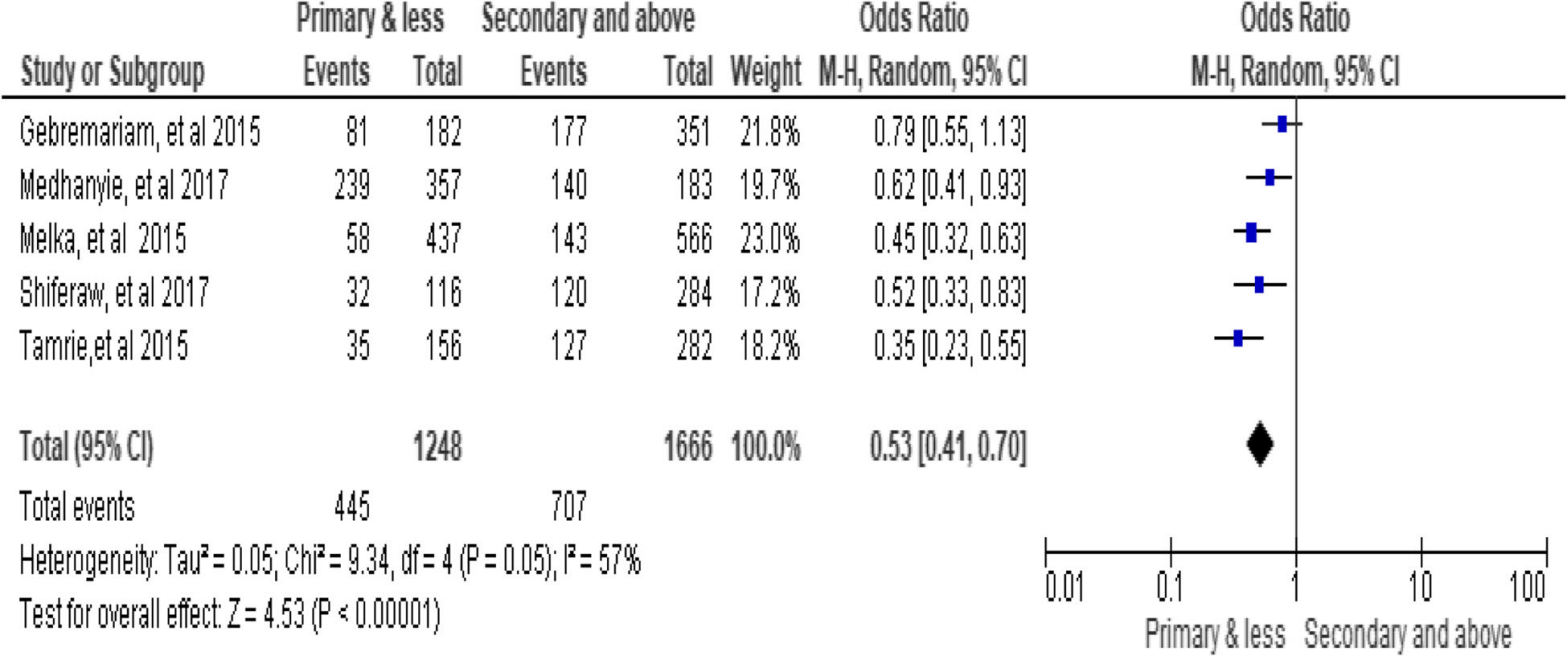
Association between husband educations with long-acting family planning service utilization in Ethiopia, 2018

The results of the analysis indicated significant association between husband’s education level and utilization of long-acting family planning services. A husband who had primary education and lesser was less likely to utilize long-acting family planning services as compared to women who attended the secondary and above education [OR = 0.53; 95% CI: 0.41, 0.70, *P* < 0.00001] (Fig. 7)*.*

### Joint husband-wife discussion

**Fig. 8:**
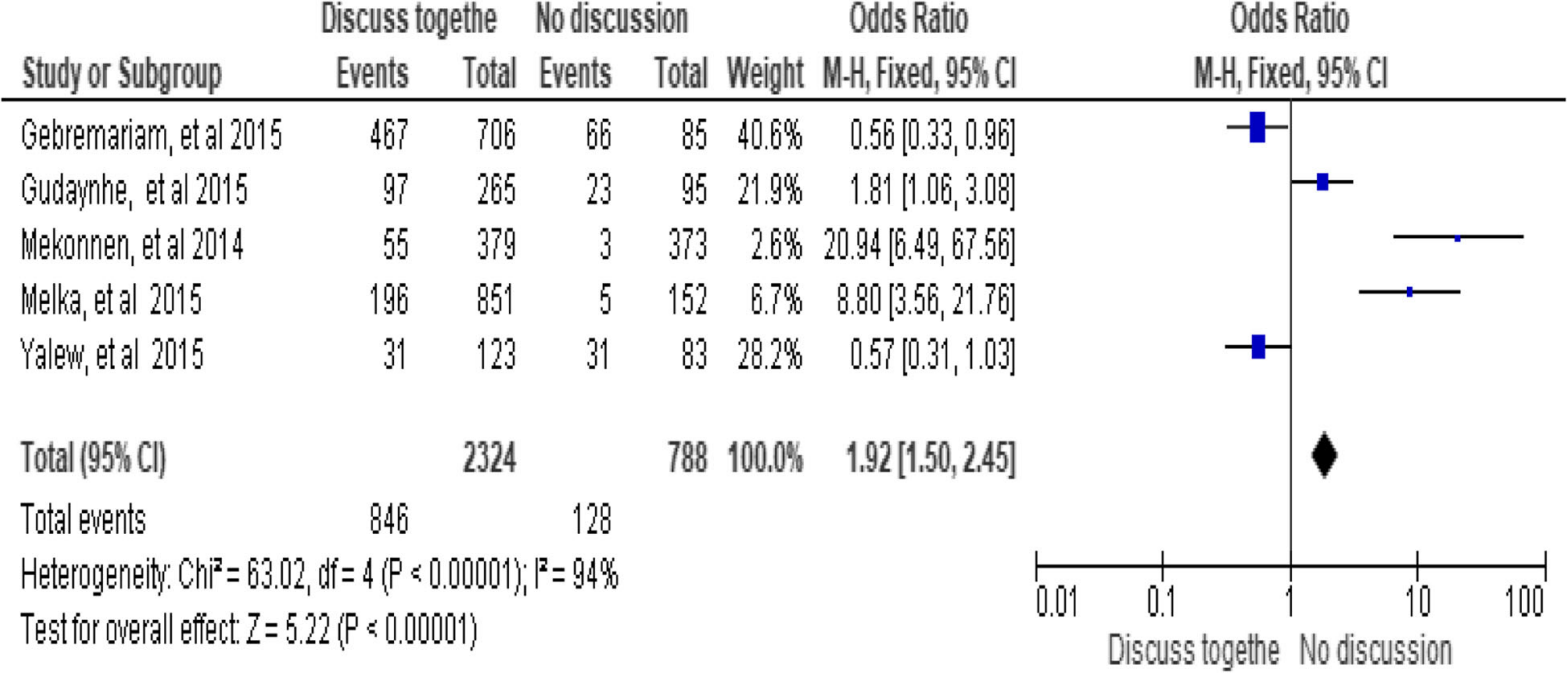
Association between husband-wife discussions and utilization of long-acting family planning services in Ethiopia, 2018

The odds ratio of the analysis indicated a significant association between husband-wife discussion and utilization of long-acting family planning services. Women who discussed about family planning methods with their partner utilize long acting family planning methods nearly two times than those who did not [OR = 1.92 (95% CI: 1.50, 2.45) P < 0.00001]. The investigators considered a fixed effect model for the analysis because the I^2^value was 94%. In addition, a Sensitivity analysis was done, and no significant change was observed in the overall summary results of odds ratio (OR) (Fig. 8)*.*

### Monthly income

**Fig. 9:**
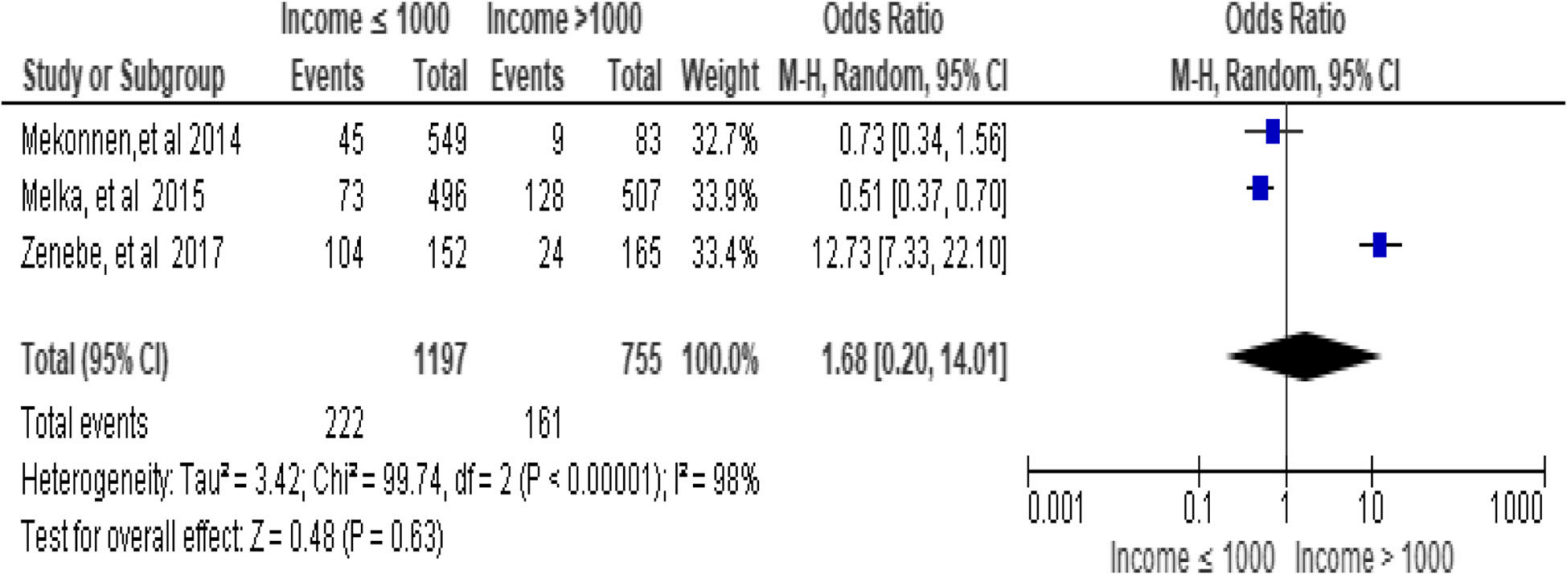
Association between income and utilization of long-acting family planning services in Ethiopia, 2018

There was no significant association between monthly income and utilization of long-acting family the planning services [OR = 1.68; 95% CI: 0.20, 14.01, *P* = 0.63]. Heterogeneity test indicated *I*^2^ = 98%, hence random effect model was assumed during analysis (Fig. 9)*.*

### Presence of electronic media

**Fig. 10:**
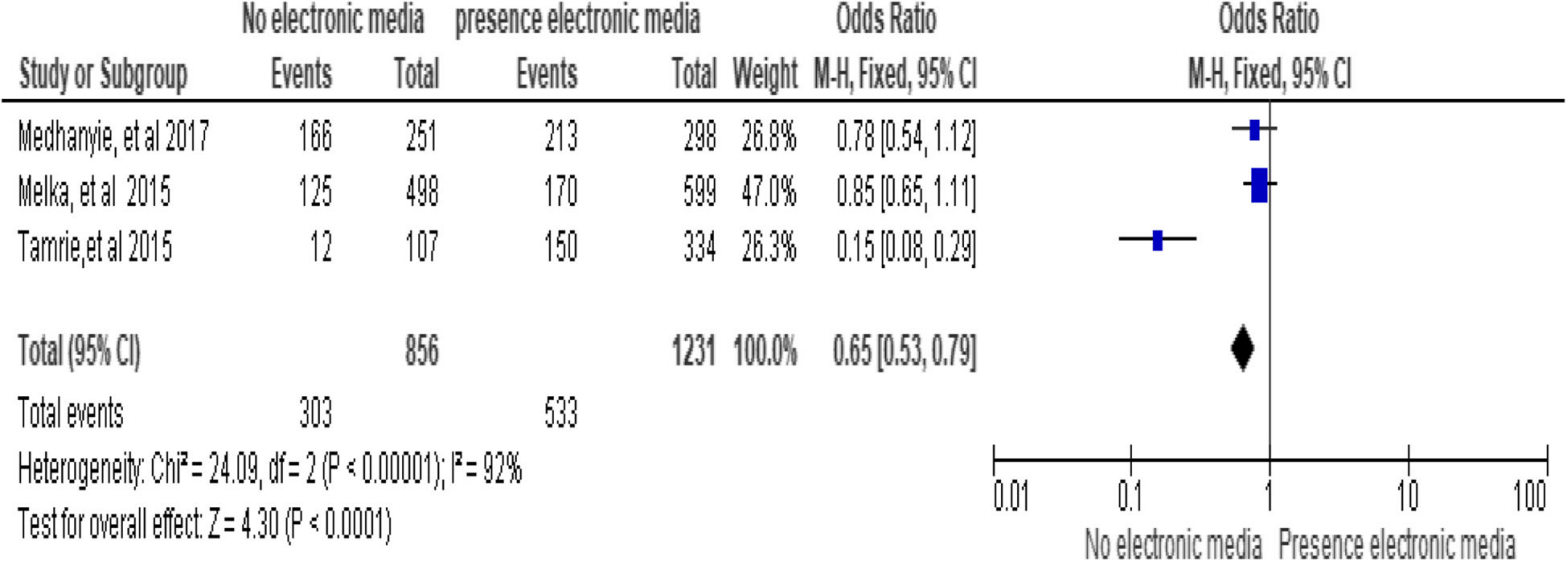
Association between presences of media with utilization of long-acting family planning services in Ethiopia, 2018

Presence of electronic media was significantly associated with utilization of long-acting family planning services. Women who do not have electronic media (radio/television) are less likely to utilize long-acting family planning services as compared to women who have electronic media [OR, 0.65; 95% CI: 0.53, 0.79, *P* < 0.0001]. Heterogeneity test indicated I^2^ = 92%, hence the fixed-effect model was assumed in the analysis. To reduce the heterogeneity, a Sensitivity analysis was done, and no change was recognized in the overall OR. In addition, the investigators applied both models interchangeably and heterogeneities were the same (Fig. 10)*.*

### Number of living children

**Fig. 11:**
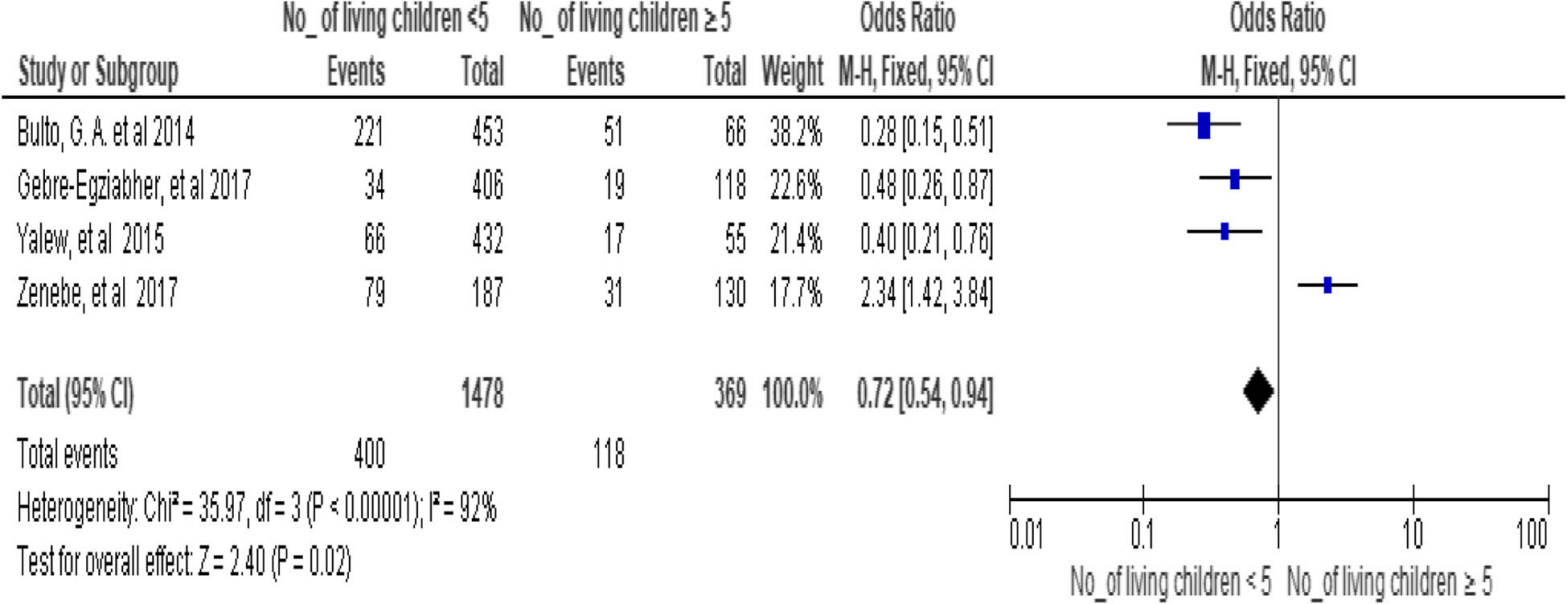
Association between numbers of living children with utilization of long-acting family planning services in Ethiopia, 2018

A significant association was found in utilizing long-acting family planning services between partners who have less than five living children and greater than or equal to five children (OR,0.72; 95% CI:0.54, 0.94, *P* = 0.02). But there was considerable heterogeneity found (I^2^ = 92%). Hence, the fixed-effect model was assumed during analysis. Here the investigators employed both models interchangeably for this analysis but no heterogeneity change was found (Fig. 11)*.*

### Previous utilization of family planning methods

**Fig. 12:**
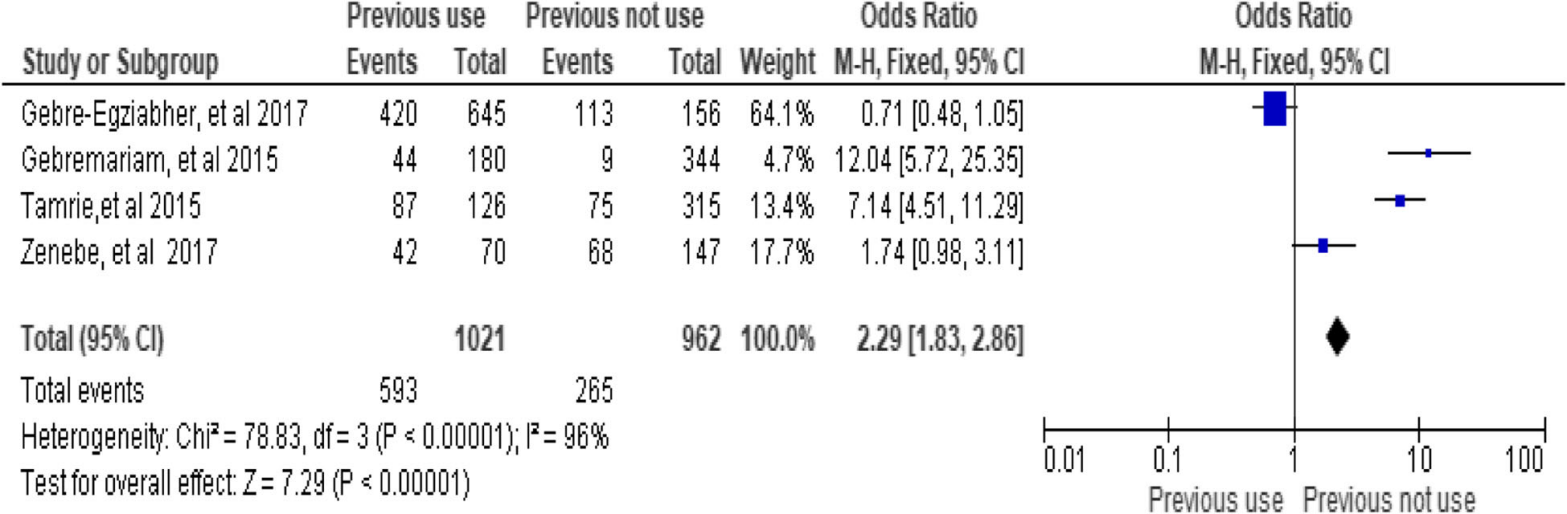
Association between previous uses of family planning method with utilization of long-acting family planning services in Ethiopia, 2018

This review demonstrated that there was no significant association between previous utilization of family planning methods and utilization of long acting family planning services(utilization) in the random model (OR, 3.16; 95% CI: 0.84, 11.85; *P* = 0.09). However, significant differences were found in the fixed effect model (OR = 2.29; 95%CI: 1.83, 2.86, *P* < 0.00001). Women who were not previously exposed to family planning use were 2.29-folds more likely to utilize long-acting family planning services as compared to women who had previous exposure. But considerable heterogeneity was found too high (I^2^ = 96%) between the studies in both models. Furthermore, significant differences were found between the two groups in the fixed effect model (Fig. 12)*.*

### Residence of the setting

**Fig. 13:**
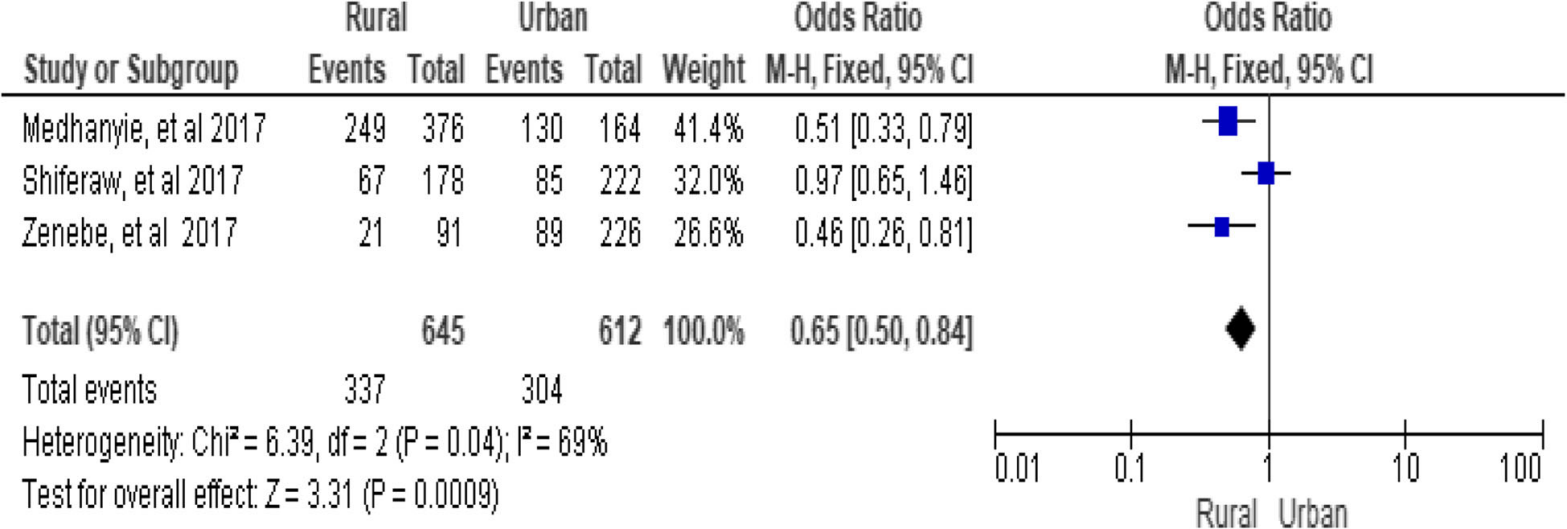
Association between residence and utilization of long-acting family planning services in Ethiopia, 2018

The residence of women was significantly associated with utilization of long-acting family planning services (utilization). Results of this review revealed that residence (as defined as rural and urban) was one of the affecting factors that determined the utilization of long-acting family planning services. Women from rural areas were less likely to use long-acting family planning services than those (women) from urban areas (OR = 0.65; 95% CI: 0.50, 0.81, *P* = 0.0009). Heterogeneity test indicated *I*^2^ = 69%, hence the fixed-effect model is assumed in this analysis because the confidence interval was very narrow (Fig. 13)*.*

**Fig. 14:**
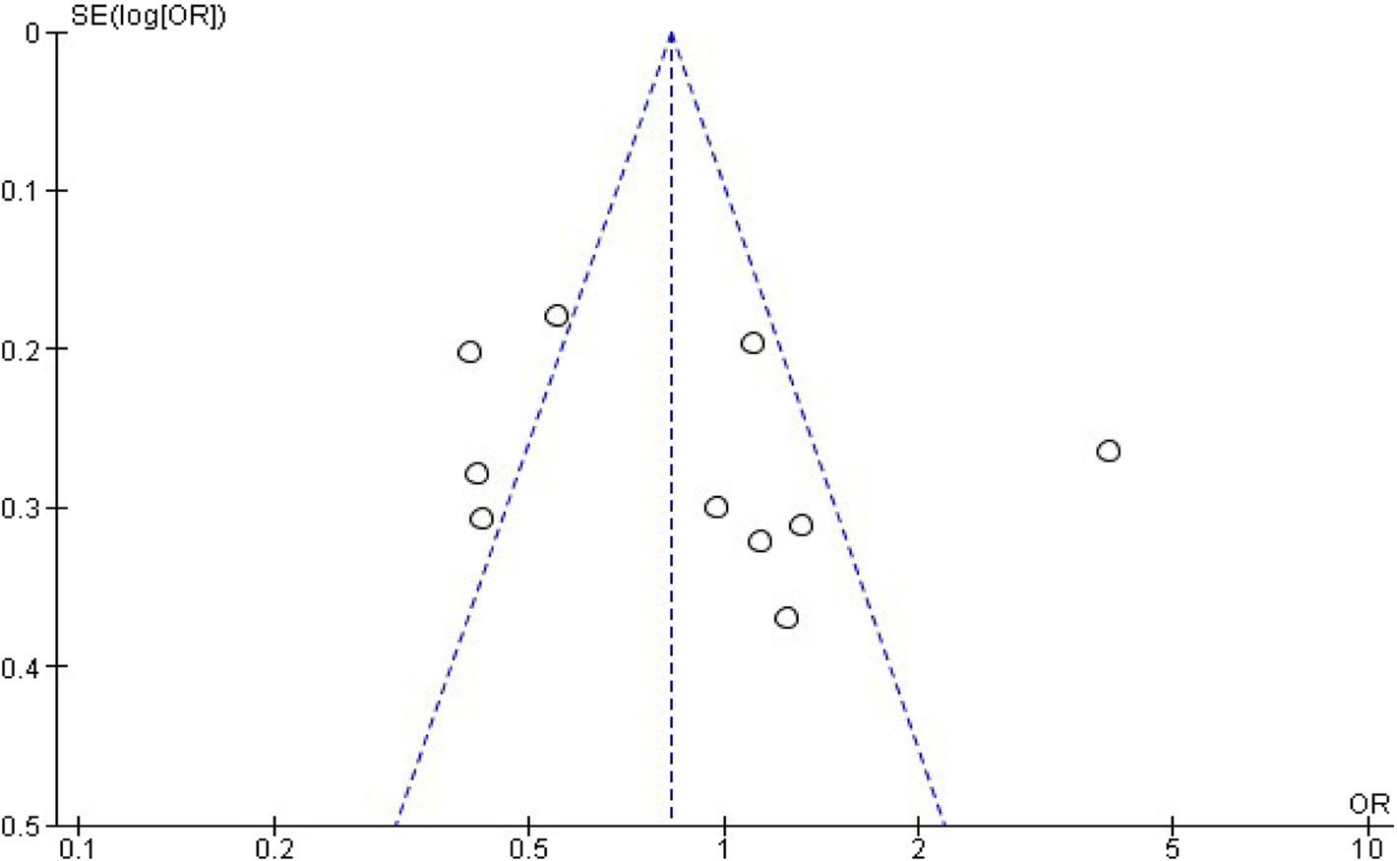
Publication bias on maternal age

**Fig. 15:**
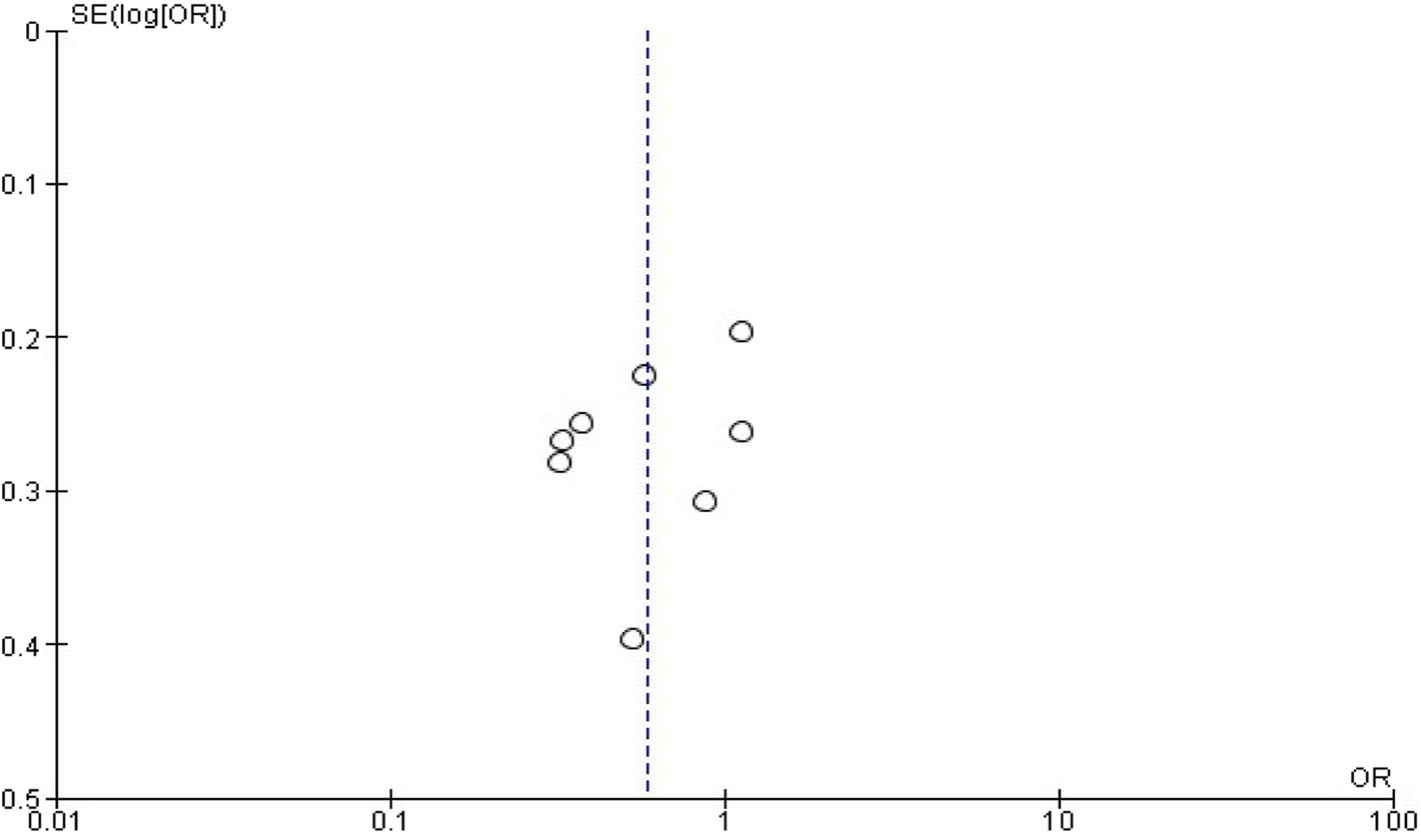
Publication bias on maternal education status

The risk of publication bias of the study presented in funnel plots (Figs. 14) and (15)*.*

## Discussion

This comprehensive study provides vibrant information of overall factors that limit utilization of long-acting family planning services in Ethiopia. In this review, a total of 15 studies done both in the community and facility-based setting in different regions of Ethiopia were included. From the included studies, 10 were community-based cross-sectional studies [8–12, 14–17, 20]. Three other studies were institutional based cross-sectional studies [19, 21, 22]; the last two studies were unmatched case-control [13, 18].

According to the Ethiopian Demographic and Health Survey Conducted in 2016, The utilization of long-acting family planning methods in Ethiopia was very poor [2]. The findings of this systematic review and meta-analysis revealed many factors that contribute to underutilization of LAFPM. Among the leading factors, the previous history of using family planning method was the main one. Women who had experience of using long-acting family planning method was not encouraged to reuse it again [10, 11, 20, 22]. It might be related to. Cultural attitudes and religious beliefs, fallacy thinking (like, it might cause infertility), inadequate knowledge about LAFPM (side effects) as well as adverse effects (amenorrhea, unscheduled bleeding patterns, weight gain etc.) contributing for not using LAFPM again, this may be associated With inadequately trained family planning counselor and do not provide the service all the times in all health institutions [24]. Attitudinal change and adequate information are the main factors that help mothers to be a user of long-acting family planning in this regard. Secondly, discussion with husband was the critical factor that limits long-acting family planning services utilization [10, 13, 15, 16, 21]. This is because of husbands are not happy in using of long-acting family planning, because they want to have more children. It needs extra work on the attitude change to alter this habit.

In addition, Being young adult women (Maternal age between 15 and 34 years), women with no formal education, husband’s education level below primary education, having less than five living children, being government- employed women, women’s husband being a government employee, having no electronic media at home, living in a rural area, and low maternal knowledge about LAFPM were among the factors contributing to the poor utilization of long-acting family planning services in Ethiopia [8–12, 14–17, 19–22]. This is comparable with the review of the Asia Pacific region [25].

### Strengths and limitations

The investigators used extensive and comprehensive search strategies from multiple databases. Published, unpublished studies and grey literature were included. Studies were evaluated for methodological quality using a standardized tool. Although the literature search was systematic and assessed all related studies within the desired scope, it is possible that relevant publications, e.g. publications reported in non-English language and local languages must have been missed. Studies with abstract were the only ones included. This may affect the finding’s inclusiveness. This study doesn’t include any findings (studies) from Benishangul-gumuz Region. This may affect the generalization of this study but still, the study will be applicable to all parts of Ethiopia.

## Conclusion

Long-acting family planning methods are underutilized in Ethiopia due to lack of information and knowledge about them. This leads women to develop a negative attitude towards these methods, the so-called joint husband-wife discussion inhibits utilization of LAFPMs. In addition, being young adult women (maternal age between 15 and 34 years), women with no formal education, husband’s education below primary level, having less than five living children, being government- employed women, women’s husband being a government employee, not having electronic media at home, and residing in a rural area were among the factors contributing to the poor utilization of long-acting family planning services in Ethiopia. Hence, the investigators suggest that key stakeholders should design interventions like behavioral change communication (BCC) and community health education through health extension workers (women discussion group in the village) to avert attitudinal and informational barriers towards long acting family planning methods.

## Additional files

Additional file 1: Preferred Reporting Items for Systematic Reviews and Meta-Analyses: The PRISMA Statement 2009 Checklist. (DOCX 19 kb)

Additional file 2: Sample search string for CINHAL and MEDLINE databases, EBSCOhost Interface. (DOCX 14 kb)

## Data Availability

The data that support the review findings of this study are available upon submitting a reasonable request to the corresponding author.

## Abbreviations

IUD: Intrauterine Device
LAFPM: Long-Acting Family Planning Methods
MeSHs: Medical Subject Headings
OR: Odds Ratio
PRISMA: Preferred Reporting Items for Systematic Reviews and Meta-analysis
