# Supplementary material for: Factors associated with long-acting family planning service utilization in Ethiopia: a systematic review and meta-analysis
Source: Contracept Reprod Med. 2019 Oct 1;4:14. doi: 10.1186/s40834-019-0095-z (PMC6771115; doi:10.1186/s40834-019-0095-z)
Supplement: Supplementary file 2 — Sample search string for CINHAL and MEDLINE databases, EBSCOhost Interface. (DOCX 14 kb) [file 40834_2019_95_MOESM2_ESM.docx]

***Additional file 2*; Sample search string for CINHAL database, EBSCOhost Interface**

| **#** | **Query** | **Limiters/Expanders** | **Last Run Via** | **Results** |
| --- | --- | --- | --- | --- |
| S4 | (Ethiopia) AND (S1 AND S2 AND S3) | Search modes - Find all my search terms | Interface - EBSCOhost Research Databases  Search Screen - Advanced Search  Database - CINAHL Complete | 37 |
| S3 | Ethiopia | Search modes - Find all my search terms | Interface - EBSCOhost Research Databases  Search Screen - Advanced Search  Database - CINAHL Complete | 3,544 |
| S2 | family planning service | Search modes - Find all my search terms | Interface - EBSCOhost Research Databases  Search Screen - Advanced Search  Database - CINAHL Complete | 6,021 |
| S1 | factors associated OR Determinants OR Predictors | Search modes - Find all my search terms | Interface - EBSCOhost Research Databases  Search Screen - Advanced Search  Database - CINAHL Complete | 286,427 |

***Additional file 2*; Sample search string for Medline database, EBSCO host Interface**

| **#** | **Query** | **Limiters/Expanders** | **Last Run Via** | **Results** |
| --- | --- | --- | --- | --- |
| S4 | (Ethiopia) AND (S1 AND S2 AND S3) | Search modes - Find all my search terms | Interface - EBSCOhost Research Databases  Search Screen - Advanced Search  Database - MEDLINE | 26 |
| S3 | Ethiopia | Search modes - Find all my search terms | Interface - EBSCOhost Research Databases  Search Screen - Advanced Search  Database - MEDLINE | 14,960 |
| S2 | family planning service | Search modes - Find all my search terms | Interface - EBSCOhost Research Databases  Search Screen - Advanced Search  Database - MEDLINE | 5,851 |
| S1 | factors associated OR Determinants OR Predicators | Search modes - Find all my search terms | Interface - EBSCOhost Research Databases  Search Screen - Advanced Search  Database - MEDLINE | 1,016,054 |

Bottom of Form
